# Keystone Veterinary Medical Association

**Published:** 1895-03

**Authors:** W. L. Rhoads

**Affiliations:** Secretary


Secretary.

### KEYSTONE VETERINARY MEDICAL ASSOCIATION.

The monthly meeting of the Keystone Veterinary Medical Association was called to order by President Lintz at the office of Dr. W. H. Hoskins, 3452 Ludlow St., Phila., Tuesday evening, February 12, with the following members present: Drs. Bridge, Eves, Hoskins, Lintz, McAnulty, McClellan, and Rhoads, with Drs. J. T. Fairly and J. O. George present as visitors.

Dr. Hoskins, as chairman of the Legislative Committee, gave a concise history of the movements and present position of the bills now before the Legislature in which all progressive veterinarians should be interested, viz.: One is to establish a live stock sanitary commission. Mr. Edge's bill and the one known as the veterinarians' bill having been modified and blended, are now one bill and known as House bill 24.

There is also a bill to establish an office of State veterinarian on the State Board of Agriculture, and another introduced by Mr. Harvey to prevent docking of horses.

The application of Dr. L. Pearson for membership was referred to the Board of Censors.

The essayist being absent, cases were next reported.

Dr. Bridge, upon being questioned, said the supposed cases of contagious pleuro-pneumonia reported at Chester Heights and Danville were bronchial trouble.

He also reported a case of tetanus in a cow from acute peritonitis, and another in a Jersey cow from puncture wound of the foot by nail.

Dr. Hoskins reported recovery from tetanus in three weeks under bromide potassium treatment.

Dr. Eves thinks if puncture wounds of the feet were immediately soaked in creoline solution tetanus would be prevented.

Dr. Lintz exhibited clamp used in fracture of infra- or supra-maxillary bone.

W. L. RHOADS,
Secretary.

